# Long-Term Survival and Improved Quality of Life following Multiple Repeat Gamma Knife Radiosurgeries for Recurrent Glioblastoma Multiforme: A Case Report and Review of the Literature

**DOI:** 10.1155/2013/431857

**Published:** 2013-09-24

**Authors:** Erik W. Larson, Halloran E. Peterson, Robert K. Fairbanks, Wayne T. Lamoreaux, Alexander R. Mackay, Jason A. Call, John J. Demakas, Barton S. Cooke, Christopher M. Lee

**Affiliations:** ^1^Gamma Knife of Spokane, Cancer Care Northwest, 910 W 5th Avenue, Suite 102, Spokane, WA 99204, USA; ^2^Cancer Care Northwest, 910 W 5th Avenue, Suite 102, Spokane, WA 99204, USA; ^3^University of Washington School of Medicine, 1959 NE Pacific Street, Seattle, WA 98195, USA; ^4^MacKay & Meyer MDs, 711 S Cowley Street, Suite 210, Spokane, WA 99202, USA; ^5^Spokane Brain & Spine, 801 W 5th Avenue, Suite 210, Spokane, WA 99204, USA

## Abstract

The management of glioblastoma multiforme (GBM) is in most cases complex and must be specifically tailored to the needs of the patient with the goals of extended survival and improved quality of life. Despite advancements in therapy, treatment outcomes remain almost universally poor. Salvage treatment options for the recurrence of the disease is an area of intense study. The following case highlights the utility of Gamma Knife Radiosurgery (GKRS) as a salvage treatment. In this clinical situation, three sequential GKRS treatments led to prolonged survival (beyond four years after diagnosis) and improved quality of life in a patient who was unable to receive further chemotherapy regimens and was unwilling to undergo further aggressive resection. To date, there have been few reports of three or more sequential GKRS treatment sessions utilized as salvage therapy for recurrent GBM in patients who can no longer tolerate chemotherapy. This report provides evidence that aggressive local treatment with GKRS at the time of recurrence may be appropriate, depending on a patient's individual clinical situation, and can lead to prolonged survival and improved quality of life.

## 1. Introduction

Glioblastoma multiforme (GBM) is the most common type of malignant primary brain tumor and is uniformly fatal [1]. The reported median survival is less than six months without treatment. Appropriately tailored multimodal treatment options including surgical resection, chemotherapy, and radiosurgery have been reported to extend median rates of survival to approximately 16 months [2]. It is known that survival rates decline with increased age at initial diagnosis. In addition, poor prognosis in these situations is in large part due to the microscopic infiltration of tumor cells into neighboring healthy brain tissue, thereby limiting the extent of resection, thus increasing the recurrence rate. Despite aggressive therapies, progressive neurologic deficits, edema, and increased intracranial pressure often result in the patient's death.

Following recurrence of GBM, treatment options are often limited and should be tailored to a patient's needs. The goal of salvage therapy is to extend longevity and maximize quality of life. Repeated surgery in most cases is too invasive or ineffective at achieving a total resection once the tumor has diffusely invaded healthy tissue. Repeated radiation therapy may not be an option due to the high cumulative risk of neurotoxicity. Gamma Knife Radiosurgery (GKRS) as a salvage treatment has shown to increase progression-free survival by 15 months when combined with chemotherapy [3]. If chemotherapy is not well tolerated, retrospective studies have shown that there can still be a significant survival benefit from GKRS treatment alone for recurrent GBM in select patients [4].

In this paper, we discuss the unique treatment results of a patient who was treated with GKRS on three separate occasions following serial repeat recurrences of GBM. At the time of this report, he is alive and being followed in a clinic for more than four years since his initial diagnosis and gross total resection.

## 2. Case Report

**Figure 1:**
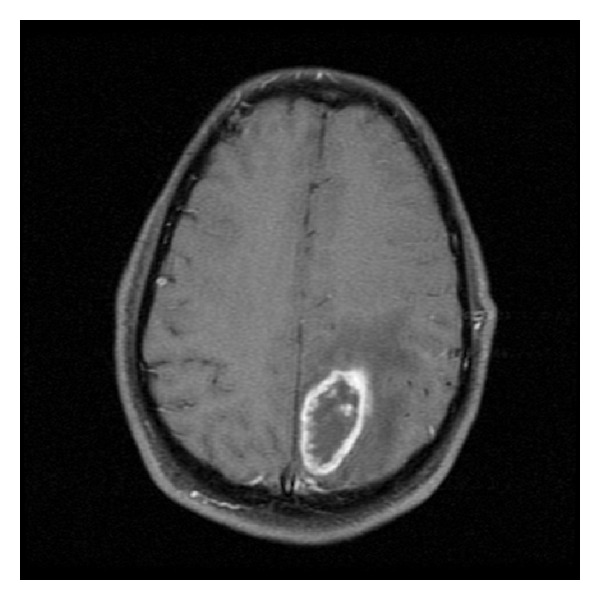
MRI scan obtained after this patient initially presented to the emergency department demonstrating 4.5 cm, ring-enhancing, left parietal mass with surrounding edema consistent with GBM.

 We report the unique case of a 46-year-old man who initially presented to the emergency department with gradually increasing headache, difficulty with sleep, fatigue and clumsiness in the right arm, and one episode of syncope. His initial MRI scan revealed a single, 4.5 cm, ring-enhancing, left parietal mass with surrounding edema which was consistent with glioblastoma multiforme (GBM) (see Figure 1). He was admitted, and a left parietal craniotomy and gross total resection of the tumor were performed. The final pathologic diagnosis confirmed that the tumor was a WHO Grade IV GBM. He recovered well from surgery with no focal neurologic deficits.

In the two months following resection, he underwent adjuvant radiation and chemotherapy. Fractionated external beam radiotherapy was administered to a dose of 60 Gy in 30 fractions over 6 weeks. Concurrent temozolomide was given at the standard dose which caused severe nausea (which necessitated treatment with antiemetics). Despite the side effects, he completed the chemotherapy regimen.

**Figure 2:**
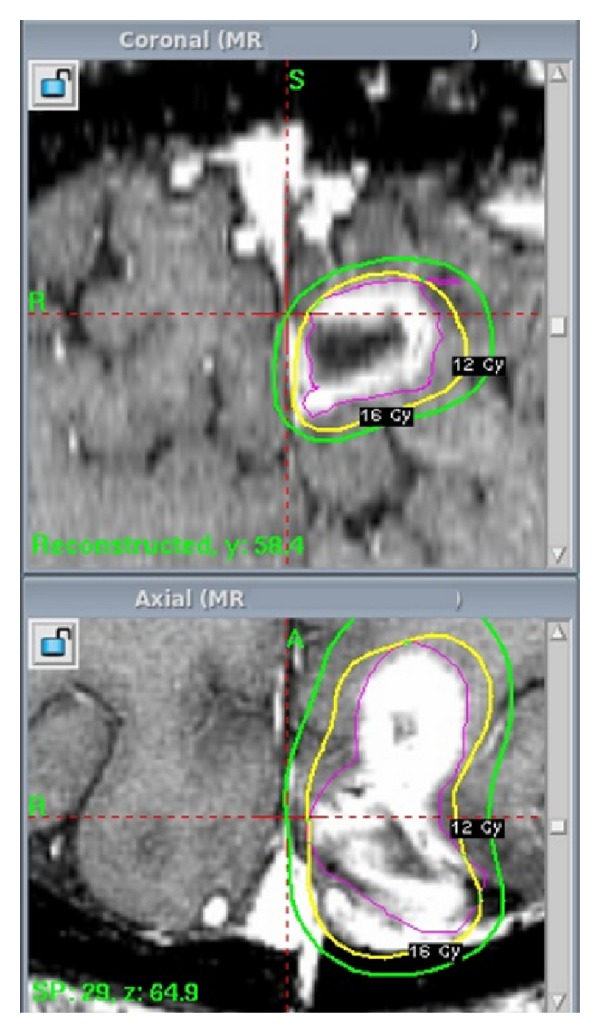
Four months after initial diagnosis, the patient suffered a recurrence of GBM and was treated with GKRS. The prescribed dose was 16 Gy to the 50% isodose line. Tumor volume was measured at 5.2 cubic centimeters.

Four months after the initial diagnosis, he began experiencing recurrent symptoms including dizziness, headaches, blurred vision, ataxia, tinnitus, some confusion, and occasional right-sided hand spasms and numbness. Repeat T1 postgadolinium MRIs revealed a new 1.5 cm nodule of tumor growing anteriorly off of the resection bed. A multidisciplinary team including a neurosurgeon and radiation oncologist concluded that this was consistent with tumor recurrence and not adverse radiation effects. The option to treat with GKRS was presented. The patient consented, and GKRS was completed without complications and was well tolerated. The marginal prescription dose was 16 Gy to the 50% isodose line. 99% of the tumor, at 5.2 cubic centimeters, received at least 16 Gy (see Figure 2). Beginning two months after this treatment, he started another course of chemotherapy. First, he received a full dose of temozolomide for two months. Upon completion, he was prescribed a combination therapy of bevacizumab and irinotecan. Eight months later, irinotecan was discontinued due to toxicity, and he continued on bevacizumab alone for the following three months until suffering a cerebrovascular accident of unknown cause resulting in right-sided weakness and numbness.

**Figure 3:**
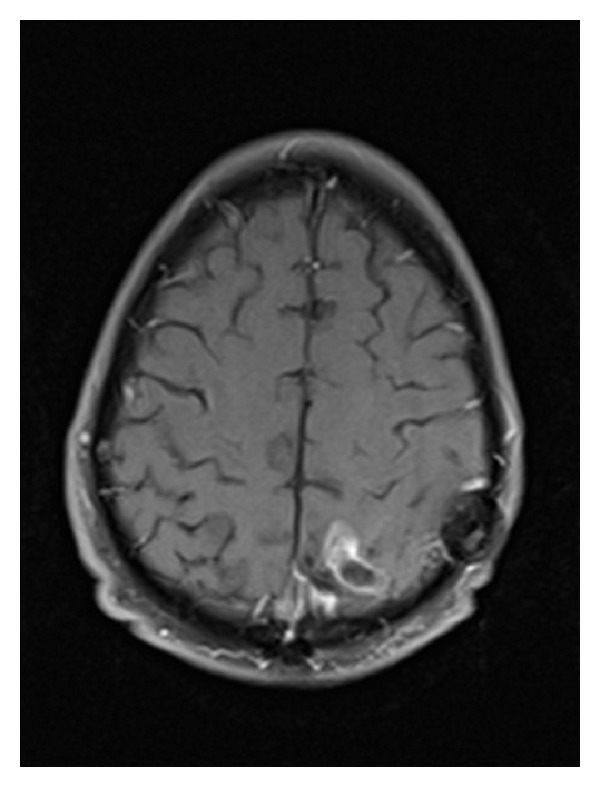
MRI scan showing increased enhancement, indicating tumor recurrence twenty-two months after GKRS salvage treatment.

**Figure 4:**
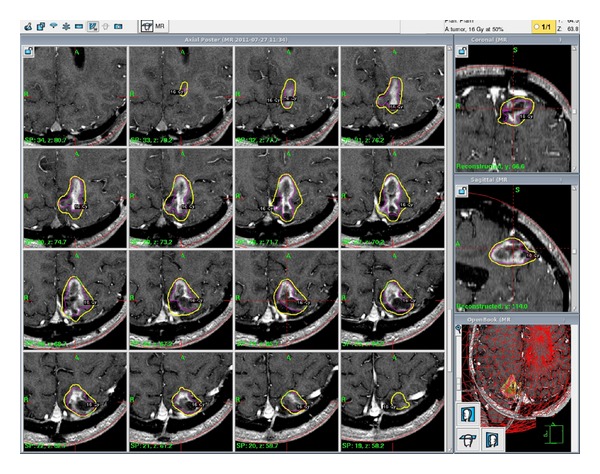
Treatment planning for second salvage GKRS treatment after the second recurrence of GBM. Again, 16 Gy was prescribed to the 50% isodose line.

 Twenty-two months after the initial GKRS treatment, the patient presented with neurologic symptoms including listing to the right, feeling off balance, reduced left-sided hearing, and a hemiparetic gait. MRI scans at 17 and 20 months after GKRS treatment revealed a new 2 cm diameter nodule deep to the resection bed with an area of enhancement in the cystic area superficial to that (see Figure 3). Comparison to the previous GKRS treatment plan demonstrated that the tumor recurrence was outside of the original treatment volume. At that time, the risks and benefits of repeat resection or repeat GKRS were discussed with the patient. The patient was interested in a repeat GKRS treatment. Once again, the prescription dose was 16 Gy to the 50% isodose line and was well tolerated by the patient (see Figure 4). He was scheduled for routine follow-up MRI scans every two months; the first of such scans showed a slight decrease in tumor size.

**Figure 5:**
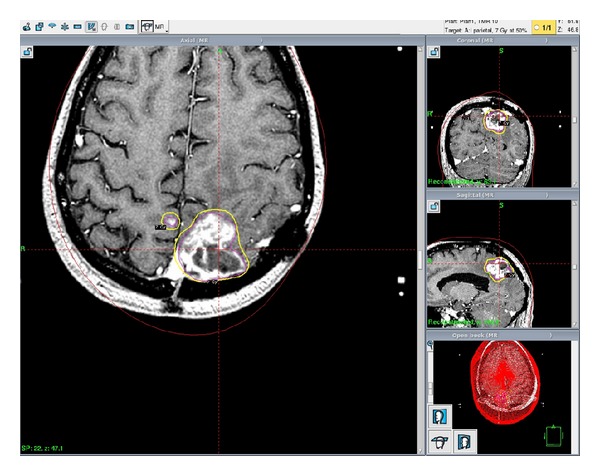
Treatment planning for one of the three fractions of 7 Gy each to the 50% isodose line (21 Gy total). This was his third and latest salvage GKRS which was administered 46 months after initial diagnosis.

 Our patient returned to the clinic twenty months after his second GKRS treatment (46 months after original gross total resection) with new complaints of decreased energy level, decreased sensation on the right side, decreased speech accuracy, blurred vision, dysdiadokinesia, and short-term memory loss. An MRI revealed an ill-defined enhancing mass and coarse calcification within the left parietal lobe along with new diffuse white matter hypodensity centered within the left parietal lobe involving the left posterior frontal lobe, posterior temporal lobe, and occipital lobe, raising suspicion for tumor progression and associated vasogenic edema. After a discussion of treatment options, fractionated GKRS was scheduled. The option of fractionation was discussed in order to limit long-term toxicity to the nearby neural structures. He received 3 separate fractions over an 8-day-period with a prescription of 7 Gy per fraction which was well tolerated (21 Gy total dose prescribed to the 50% isodose line, Figure 5).

At the present time, it has been three months since he received the fractionated GKRS treatment. MRI shows no tumor progression. While he requires the use of a wheelchair and is experiencing some fatigue and weakness following the treatment, he is not suffering from headaches, has no issues with speech, communicates well, and is living at home. 

## 3. Discussion

For newly diagnosed GBM, the best known prognosis indicators are Karnofsky Performance Score (KPS), patient's age, neurologic function, and extent of resection [5]. An appropriately tailored, multimodal treatment plan is known to statistically extend survival for a subset of patients, in particular those under the age of 50 with KPS > 60 [6–9]. For initial management of GBM, a maximal safe surgical resection is indicated. Following histopathologic analysis of a tumor sample and confirmation of the GBM diagnosis, adjuvant radiation and chemotherapy are the standard of care. Regardless of Recursive Partitioning Analysis (RPA) status and treatment approach, GBM will recur after approximately 36 weeks at the original tumor location or at a distant site within the brain in almost all patients with no further treatment. Presumably, the natural history of recurrent GBM is similar to that of its initial presentation: without treatment, median survival following recurrence is less than six months [10]. Following this inevitable recurrence, survival outcomes have been improved through various salvage treatments such as repeat surgeries, radiation therapy, and chemotherapy.

Some institutions have studied the survival benefit of repeat surgical resections of recurrent GBM. In the largest study of multiple resections to date, Chaichana and colleagues compared survival outcomes for patients receiving 1, 2, 3, or 4 resections. While controlling for known survival indicators, they reported significantly improved median overall survival rates for patients who underwent multiple resections: 6.8, 15.5, 22.4, and 26.6 months, respectively. Although very aggressive therapy, they recommend that patients who are able to tolerate the surgery should be offered repeat resection as a treatment option [7]. These repeated resections may improve survival in eligible patients by reducing tumor burden, prolonging time to recurrence, and possibly allowing for increased efficacy of adjuvant therapy [7, 8]. Additionally, for tumors which are distant from eloquent brain areas, an extended resection (1-2 cm beyond tumor border) may significantly prolong time before local recurrence, thereby extending survival [11]. These benefits, while significant, have intrinsic selection bias [6, 7]. Patients healthy enough for surgery and with accessible tumors are eligible for greater extent of resection.

 Using GKRS alone or in conjunction with surgery for salvage treatment of recurrent GBM can significantly improve survival. Although limited data is available, a 2012 retrospective study of 77 patients showed that the median post-treatment survival was 12 months for those receiving GKRS compared to 6 months for patients treated with surgery alone. Complications from the two treatment modalities were also significantly different: 9.8% for GKRS and 25.2% for reoperation [12].

The timing of GKRS for GBM is important. There does not appear to be a significant benefit when it is used as a component of adjuvant treatment for initial diagnosis of GBM, but there is a potential survival advantage in select cases when GKRS is used as salvage when GBM recurs [13, 14]. Hsieh et al. demonstrated that salvage GKRS treatment at the time of tumor progression extends median survival to 16.7 months, compared with 10 months for patients treated with adjuvant GKRS at the time of initial resection [15]. Koga et al. recently demonstrated 24 months median overall survival in patients receiving salvage GKRS at a dose of 20 Gy [16]. They also showed improved local tumor control when extending the treatment margin by 0.5–1 cm beyond the enhancing region which resulted in greater local tumor control but also an increase in adverse radiation effects [16].

 Despite its specific targeting capability and sharp dose falloff, GKRS is not without these potential adverse effects. Acute side effects may include edema which can cause or exacerbate existing neurological symptoms. This edema typically responds to corticosteroids [17]. Longer term effects, such as brain injury or radionecrosis, may need to be treated surgically. However, it can be challenging to distinguish between a radiation effect and tumor recurrence on MR imaging alone and may require MR spectroscopy or PET. Diagnostic techniques are emerging, such as “T1/T2 mismatch,” described by Kano et al. to identify radiation effect with 83% sensitivity and 93% specificity [18].

 Chemotherapeutic agents are also being studied in the management of recurrent GBM. The landmark randomized trial using adjuvant temozolomide combined with radiation therapy showed significant progression-free survival and overall survival benefits, setting the standard of care [19]. Other institutions are exploring the safety and efficacy of adding bevacizumab, an antiangiogenic agent [20]. In a phase II trial, bevacizumab plus temozolomide for patients with recurrent disease was shown to increase progression-free survival by approximately 6 months with no benefit to overall survival in newly diagnosed GBM [21]. A small retrospective study by Cecchi et al. evaluated the use of bevacizumab in combination with irinotecan for recurrent GBM. The median progression-free survival in the combination therapy group was 15.4 months compared to 5.1 months for those receiving bevacizumab alone [22]. In a 2012 study by Park et al., selected patients experienced 33.2 months of overall survival when receiving salvage GKRS at 16 Gy followed by bevacizumab compared to 26.7 months for the cohort which did not receive bevacizumab [3].

## 4. Conclusion

This report highlights the survival and quality of life benefits of successful salvage treatment for recurrent GBM using appropriate multimodal therapy. This is one of few reported cases of a patient receiving salvage GKRS treatment on three separate sessions. He elected to undergo repeat GKRS instead of repeated open resections. More than four years after his initial diagnosis, this patient is alive and enjoying an acceptable quality of life at home with family.
